# Longitudinal Quantitative Pupillometry and Its Association with the Best Documented Level of Consciousness During ICU Hospitalization: A Single-Center Retrospective Cohort Study

**DOI:** 10.3390/diagnostics16213483

**Published:** 2026-10-24

**Authors:** Yasuyoshi Kai, Junko Yamaguchi, Toru Hosokawa, Kosaku Kinoshita

**Affiliations:** Division of Emergency and Critical Care Medicine, Department of Acute Medicine, Nihon University School of Medicine, 30-1 Oyaguchi Kamimachi, Itabashi-ku, Tokyo 173-8610, Japanhosokawa.toru@nihon-u.ac.jp (T.H.); kinoshita.kosaku@nihon-u.ac.jp (K.K.)

**Keywords:** disorders of consciousness, quantitative pupillometry, neurological prognostication, neuromonitoring

## Abstract

**Background/Objectives:** Quantitative pupillometry objectively assesses neurological status and has demonstrated prognostic value after cardiac arrest. Nonetheless, it remains unclear whether longitudinal changes in quantitative pupillary parameters are associated with concurrent trajectories of consciousness recovery during the acute phase of intensive care unit (ICU) hospitalization in comatose patients with heterogeneous underlying etiologies. We investigated associations between longitudinal quantitative pupillary trajectories and recovery of consciousness. **Methods:** This single-center retrospective cohort study included 126 adult ICU patients with Glasgow Coma Scale scores ≤8 who underwent serial constriction velocity (CV) and percentage change in pupil diameter (%CH) measurements during the first 5 ICU days. Neurological outcomes were classified into Good (minimally conscious state or better) and Bad (unresponsive wakefulness syndrome or coma) groups. Longitudinal changes were analyzed using generalized linear mixed models. **Results:** The Good outcome group consistently had higher CV and %CH than the Bad outcome group. Generalized linear mixed models showed significant group and time effects on bilateral CV and %CH, with significant group-by-time interactions only for bilateral %CH. %CH continued increasing in the Good outcome group but plateaued after Day 3 in the Bad outcome group. **Conclusions:** Serial quantitative pupillometry revealed a concurrent longitudinal association between changes in quantitative pupillary parameters and neurological status in a heterogeneous cohort of comatose ICU patients. These findings suggest that longitudinal assessment of quantitative pupillary parameters may complement multimodal neurological evaluations by providing objective information reflecting the ongoing process of consciousness recovery and dynamic changes in neurological status during the acute phase.

## 1. Introduction

Early prediction of the recovery of consciousness in comatose intensive care unit (ICU) patients is essential for clinical decision-making, family counseling, and neurocritical care management, particularly in patients with heterogeneous underlying etiologies. Despite advances in multimodal neuroprognostication, reliable methods for the early prediction of consciousness recovery in heterogeneous ICU populations remain limited [1,2,3]. Neurological examinations have traditionally relied on the assessment of pupil size and the pupillary light reflex to detect neurological deterioration, including brain herniation [4].

Nevertheless, manual visual assessment is limited by substantial inter-observer variability and the inability to detect or quantify subtle changes in pupillary responses [5]. The introduction of infrared-based automated pupillometers has enabled objective, reproducible, and quantitative assessment of pupillary function [4,6]. Previous studies have demonstrated the prognostic utility of quantitative pupillometry in selected neurological conditions, particularly post-cardiac arrest syndromes [7,8]. Nonetheless, most investigations have focused on disease-specific populations or evaluated pupillary parameters at a single time point during hospitalization [9]. Consequently, little is known about the longitudinal trajectories of quantitative pupillary responses or their relationship with consciousness recovery in heterogeneous cohorts of comatose ICU patients.

This study aimed to investigate whether quantitative pupillary parameters, including percentage change in pupil diameter (%CH) and constriction velocity (CV), and their longitudinal changes measured using automated pupillometry were associated with the best documented level of consciousness achieved during ICU hospitalization in comatose ICU patients. We hypothesized that longitudinal changes in quantitative pupillary parameters would be associated with this retrospectively classified level of consciousness.

## 2. Materials and Methods

### 2.1. Study Design and Patients

This single-center retrospective observational cohort study used hospital database and patients’ electronic medical record data. It was approved by the Institutional Review Board and Ethics Committee of Nihon University Itabashi Hospital (approval no. RK-250909-1), and informed consent was waived because of its retrospective nature.

This retrospective observational study included adult patients (aged ≥ 18 years) who were admitted to the intensive care unit (ICU) between January 2024 and December 2025 and had a Glasgow Coma Scale (GCS) score of ≤8 at ICU admission. Patients were excluded if they had pre-existing structural pupillary abnormalities, died within five days of ICU admission, or had no quantitative pupillometry measurements recorded on one or more study days during their individual observation periods. The study was based on data collected during routine ICU care rather than according to a prospective research protocol. In routine clinical practice, patients with severe physiological instability and a high risk of imminent death, including those with post-cardiac arrest syndrome following prolonged cardiac arrest, extensive cerebral ischemic injury, or severe traumatic brain injury, were primarily managed with a focus on essential clinical interventions, nursing care, and opportunities for family visitation. Consequently, frequent quantitative pupillometry was not always prioritized in these patients, resulting in limited availability of serial measurements. Some patients also exhibited bilaterally dilated and nonreactive pupils at the initial clinical assessment. Therefore, patients who died within five days of ICU admission were excluded from the study population because of the limited availability of longitudinal pupillometry data in this subgroup.

### 2.2. Pupillometry Measurement and Data Collection

As part of routine clinical care, quantitative pupillary measurements were performed every 2 h by trained ICU nursing staff using a portable automated pupillometer (NPi-300; NeurOptics Inc., Irvine, CA, USA) [10].

For longitudinal data collection, the device was equipped with a patient-specific SmartGuard^®^ (SG-200; NeurOptics Inc., Irvine, CA, USA) attachment containing an embedded radio-frequency identification (RFID) chip, enabling automatic patient identification and electronic storage of serial pupillometry measurements. The devices used during the study period operated with firmware versions 1.00–3.00.

#### 2.2.1. Device Operation and Measurement Technique

For each measurement, the operator positioned the NPi^®^-300 pupillometer equipped with the SmartGuard^®^ attachment perpendicular to the patient’s visual axis to minimize measurement artifacts and device tilt. The device position was then adjusted to center the pupil on the liquid crystal display (LCD) screen. Proper alignment and focal distance were confirmed when the tracking box surrounding the pupil turned green. Once the green tracking box was displayed, the operator pressed the measurement button and held the device steady for approximately 3 s for each eye. During this period, the device delivered a standardized light stimulus and recorded the dynamic pupillary response frame by frame, allowing quantitative pupillary parameters to be calculated automatically. According to the manufacturer’s operating instructions available to us, no user-operated manual calibration procedure was specified for the NPi^®^-300 pupillometer. Accordingly, no manual calibration was performed by the clinical staff during the study period.

#### 2.2.2. Clinical Measurement Protocol and Data Handling

All pupillometry measurements were performed by the ICU nurses responsible for each patient’s care as part of routine nursing practice. Although no study-specific training was provided, all participating nurses had received instruction in the standardized operating procedures for the NPi^®^-300 pupillometer through routine clinical training.

Measurements were initiated as part of the initial assessment upon ICU admission and were subsequently performed approximately every two hours throughout the 24 h period, with both eyes consistently assessed whenever clinically feasible. The actual measurement intervals occasionally varied because of clinical procedures, diagnostic imaging, surgery, or other circumstances encountered during routine ICU care.

A single measurement was obtained from each eye at each scheduled assessment, and routine repeated measurements at the same time point were not performed. If the device displayed an error, the measurement was repeated in an attempt to obtain valid reading. The first valid measurement obtained at each assessment was used for the analysis.

When measurements could not be obtained because of device detection or resolution limitations, such as extremely small pupils (pupil diameter < 0.80 mm), and repeated attempts remained unsuccessful, the corresponding observations were recorded as measurement errors and treated as missing data in subsequent statistical analyses.

Measurements from the right and left eyes were analyzed separately as distinct variables, without averaging or otherwise combining the bilateral measurements.

Although the NPi^®^-300 pupillometer is designed to minimize the influence of ambient illumination on quantitative pupillary measurements, attention was also paid to the lighting conditions during routine clinical assessments. Ambient lighting in the ICU followed the institutional day–night lighting schedule, with room lights maintained during the daytime (07:00–21:00) and turned off during the designated nighttime period (21:00–07:00).

To summarize repeated pupillometry measurements within each calendar day and account for potential within-day variability, daily mean values were calculated and used as representative values for each pupillary parameter (e.g., %CH and CV). Daily means were calculated from all available valid measurements obtained within each calendar day (00:00–24:00), provided that at least one valid measurement was available. For days with incomplete 24 h coverage due to nighttime ICU admission, ICU discharge, or interruptions in clinical care, all available valid measurements were similarly averaged. The period from 07:00 to 21:00 represented the standard daytime lighting schedule in the ICU and was not used to restrict the measurement or analysis window.

In this study, serial quantitative pupillometry data obtained during routine ICU care were retrospectively reviewed. Pupillometry was performed from ICU admission until recovery of consciousness to the presumed premorbid level, ICU discharge, or the end of the clinically indicated monitoring period, whichever occurred first. The duration of pupillometry monitoring varied according to each patient’s clinical course, ranging from a few days to 14 days or longer.

For the assessment of neurological outcomes, the observation period was defined as the interval from ICU admission to the assessment at which the best neurological status was documented in patients classified as having emerged from the minimally conscious state (EMCS), being in a minimally conscious state (MCS), or having unresponsive wakefulness syndrome (UWS). For patients who remained in a coma, the observation period extended to the time of the final neurological assessment.

The primary longitudinal analysis was restricted to the first five days following ICU admission (Days 1–5). This period was selected because it represents the early acute phase, during which therapeutic interventions are generally most intensive, and because the number of patients with available pupillometry data decreased substantially after Day 5, limiting the feasibility of reliable longitudinal analysis with an adequate sample size. Accordingly, pupillometry measurements obtained beyond the first five days were not included in the primary longitudinal analysis. The primary longitudinal analysis was performed using a mixed-effects model based on patient-level daily mean pupillometry values to accommodate unbalanced repeated-measures data arising from differences in the number of observation days among patients.

### 2.3. Analgesia and Sedation Protocol

Mechanically ventilated patients received analgesia and sedation according to the institutional standard protocol using an analgesia-first sedation strategy [11,12].

Remifentanil was administered as the primary analgesic agent by continuous intravenous infusion at a standard dose of 0.025 μg/kg/min. Sedatives were administered only when clinically indicated for the management of agitation or other therapeutic requirements. Propofol (0.17–3.33 mg/kg/h) or midazolam (0.017–0.33 mg/kg/h) was administered by continuous intravenous infusion and titrated to maintain a target Richmond Agitation-Sedation Scale (RASS) score between −2 and 0 [13]. Sedation depth was assessed using the RASS, according to the institutional clinical protocol [11].

Sedative and analgesic medications were generally administered by continuous intravenous infusion rather than intermittent bolus administration.

Infusion rates were adjusted according to clinical requirements and independently of the timing and results of automated pupillometry measurements. Pupillometry results were not used to guide sedation or analgesic management.

### 2.4. Neurological Outcome Assessment

The Coma Recovery Scale—Revised (CRS-R) is a standardized neurobehavioral assessment tool designed to evaluate the level of consciousness and recovery in patients with severe disorders of consciousness. One of its major advantages is its ability to distinguish among clinically relevant states of consciousness, allowing more refined classification than the Glasgow Coma Scale (GCS) [14].

Based on the findings of Boiden et al. regarding the correspondence between GCS scores and CRS-R classifications [15], we retrospectively categorized patients into four states of consciousness—coma, unresponsive wakefulness syndrome (UWS), minimally conscious state (MCS), and emergence from the minimally conscious state (EMCS)—using study-specific GCS-based operational criteria supplemented by documented clinical behavioral responses (Table 1) [15,16]. These classifications were not based on direct administration of the standardized CRS-R.

Two physicians independently reviewed the medical records and classified each patient’s level of consciousness according to the study-specific operational criteria presented in Table 1, without access to the quantitative pupillometry results. The final classification was determined by consensus following discussion between the two physicians.

Neurological outcomes were determined retrospectively based on the best documented neurological state attained during the ICU observation period, as identified through serial GCS assessments and documented clinical findings, rather than neurological status at a fixed time point during the first five days or long-term functional outcomes after hospital discharge.

Patients classified as EMCS or MCS were assigned to the Good outcome group, whereas those classified as UWS or coma were assigned to the Bad outcome group. Longitudinal changes in quantitative pupillary parameters were subsequently compared between these two groups.

The time to the best observed neurological state or final assessment was defined as the interval from ICU admission to the assessment at which the best documented neurological state was observed in patients classified as EMCS, MCS, or UWS. For patients who remained in a coma, this variable represented the interval from ICU admission to the final neurological assessment. This variable was not intended to represent time to recovery of consciousness in all patients.

Secondary objectives included examining longitudinal differences in pupillary parameters according to underlying disease category and evaluating their associations with clinical outcomes. Based on final clinical diagnoses established by board-certified specialists in emergency medicine, intensive care medicine, neurosurgery, and neurology, patients were classified into three etiological categories: structural brain injury, post-cardiac arrest brain injury, and nonstructural brain injury. For etiological classification, two authors, both board-certified specialists in emergency and critical care medicine, jointly reviewed the clinical data, neuroimaging findings, and laboratory results to determine whether the identified underlying disease or condition was the primary cause of each patient’s impaired consciousness. Each patient was subsequently assigned to one of the three etiological categories based on consensus between the two authors. Associations between pupillary parameters and clinical indicators, including the time to the best observed neurological state or final assessment, were also evaluated.

### 2.5. Statistical Analysis

Statistical analyses were performed using JMP Student’s Edition version 19 (SAS Institute, Cary, NC, USA). Statistical significance was set at a two-sided *p*-value < 0.05. Given the retrospective observational design, no a priori sample size calculation or statistical power analysis was performed. The sample size was determined by the number of patients who met the eligibility criteria during the predefined study period, and all eligible patients were included in the analysis.

Baseline characteristics were compared between Good and Bad outcome groups using chi-square tests for categorical variables and Mann–Whitney U or Kruskal–Wallis tests for continuous variables, as appropriate.

In the primary analysis, longitudinal changes in quantitative pupillometry parameters, including CV and %CH, were examined over the first 5 days after ICU admission. Generalized linear mixed models (GLMMs) assuming a Gaussian distribution with an identity link function were used to account for within-patient correlations arising from repeated measurements, with CV or %CH specified as the dependent variable [17,18].

The models included neurological outcome (good outcome [EMCS/MCS] vs. poor outcome [UWS/coma]), time (Days 1–5), and the interaction between neurological outcome and time as fixed effects. The outcome-by-time interaction was used to assess whether longitudinal changes in quantitative pupillometry parameters differed between the neurological outcome groups. To account for repeated measurements within the same patient, patient ID was included as a random intercept. Time (Days 1–5) was modeled as a categorical variable rather than as a continuous linear term, allowing for potentially nonlinear changes in pupillary parameters over time. An unstructured (UN) covariance structure was specified for the repeated measurements, and model parameters were estimated using restricted maximum likelihood (REML). Fixed-effect parameter estimates (β coefficients) and their corresponding 95% confidence intervals (CIs) were reported to describe the magnitude and precision of the estimated associations.

Incomplete longitudinal observations were accommodated within the mixed-model framework without excluding patients solely because of missing measurements at individual time points. Under the assumption that data were missing at random (MAR), the likelihood-based estimation approach incorporated all available observations without requiring complete data across all five assessment days. Although the primary mixed-model analysis assumed that data were missing at random (MAR), missing observations may have been informative and potentially missing not at random (MNAR), as they were associated with clinical trajectories, including early ICU discharge following neurological recovery and treatment limitation in patients with clinical deterioration. To assess the consistency of the primary findings among patients with complete longitudinal measurements, an additional sensitivity analysis was performed by restricting the analysis to patients with complete representative measurements across all five observation days. To assess the potential influence of clinical confounding factors on the longitudinal associations examined in this study, additional prespecified sensitivity analyses were performed using generalized linear mixed models (GLMMs). These models were adjusted for baseline characteristics, including age, sex, and underlying etiology, as well as treatment-related factors, such as targeted temperature management (TTM) in patients following cardiac arrest.

To evaluate potential medication-related confounding, additional sensitivity analyses were performed using GLMMs separately adjusted for continuous sedative administration and continuous analgesic administration. Medication exposure was represented by binary covariates indicating the presence or absence of continuous infusion. Detailed time-varying medication doses and cumulative drug exposure were not incorporated into the models.

Furthermore, to account for neurological severity at ICU admission, the Glasgow Coma Scale (GCS) score was incorporated into the models. As all patients were intubated at ICU admission, precluding reliable assessment of the verbal response component (V), the sum of the eye-opening (E) and motor response (M) scores was used for adjustment.

Separate GLMM analyses were performed for the three predefined etiological subgroups: structural brain injury, post-cardiac arrest brain injury, and non-structural brain injury. Finally, given the limited sample sizes in certain subgroups, all etiology-specific subgroup analyses were considered strictly exploratory and hypothesis-generating.

Model adequacy was evaluated using graphical diagnostics of conditional residuals from the four models for right- and left-eye %CH and CV. Normal quantile–quantile (Q–Q) plots and residual-versus-fitted-value plots were visually inspected to assess departures from normality, potential heteroscedasticity, and systematic residual patterns.

Post hoc pairwise comparisons used Tukey’s multiple-comparison test.

## 3. Results

### 3.1. Patient Characteristics

**Figure 1:**
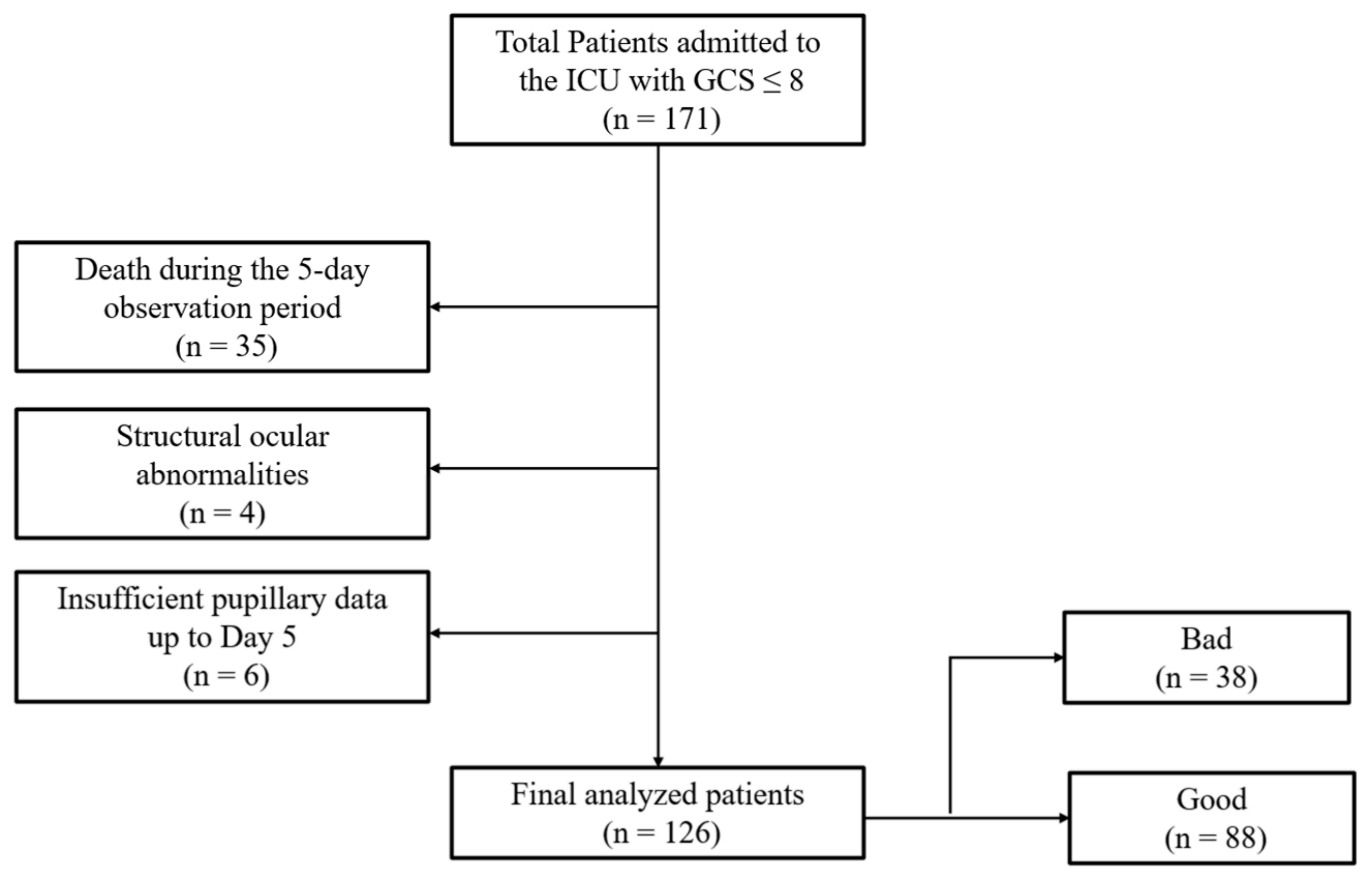
Study flow diagram of patients included in the analysis (n = 126). Abbreviations: Good, Good outcome group (CRS-R), which included cases with EMCS and MCS; Bad, Bad outcome group, which included cases with UWS and coma.

Of 171 patients who met the initial eligibility criteria during the study period, 45 were excluded: 35 died within 5 days of ICU admission, four had pre-existing structural ocular abnormalities, and six had insufficient pupillometry data during the first 5 days. The final analysis included 126 patients (Figure 1).

By neurological outcome, 88 (69.8%) and 38 (30.2%) patients were classified into the Good and Bad outcome groups, respectively. Baseline characteristics are summarized in Table 2. The median age of the overall cohort was 70.5 years, and 75 patients (59.5%) were male. Patients in the Bad outcome group were significantly older than those in the Good outcome group (median age, 77 vs. 65 years, *p* = 0.0143).

The numbers of patients contributing data on each study day, the number of pupillometry measurements per patient, and the reasons for missing data and dropout among the 126 patients included in Table 2 were summarized by neurological outcome group in (Supplementary Table S1). Of the 126 patients, 27 (21.4%) did not have complete data for the first five study days, primarily because of ICU discharge or transfer to a general ward following clinical improvement (n = 23) or decisions to withhold further treatment due to clinical deterioration (n = 4).

Underlying etiologies differed significantly between groups (*p* = 0.0098). Post-cardiac arrest brain injury was more frequent in the Bad outcome group than in the Good outcome group (26.3% vs. 10.2%), whereas nonstructural brain injury was more common in the Good outcome group (35.2% vs. 13.2%). Structural brain injury frequency was similar between groups (Table 2).

**Figure 2:**
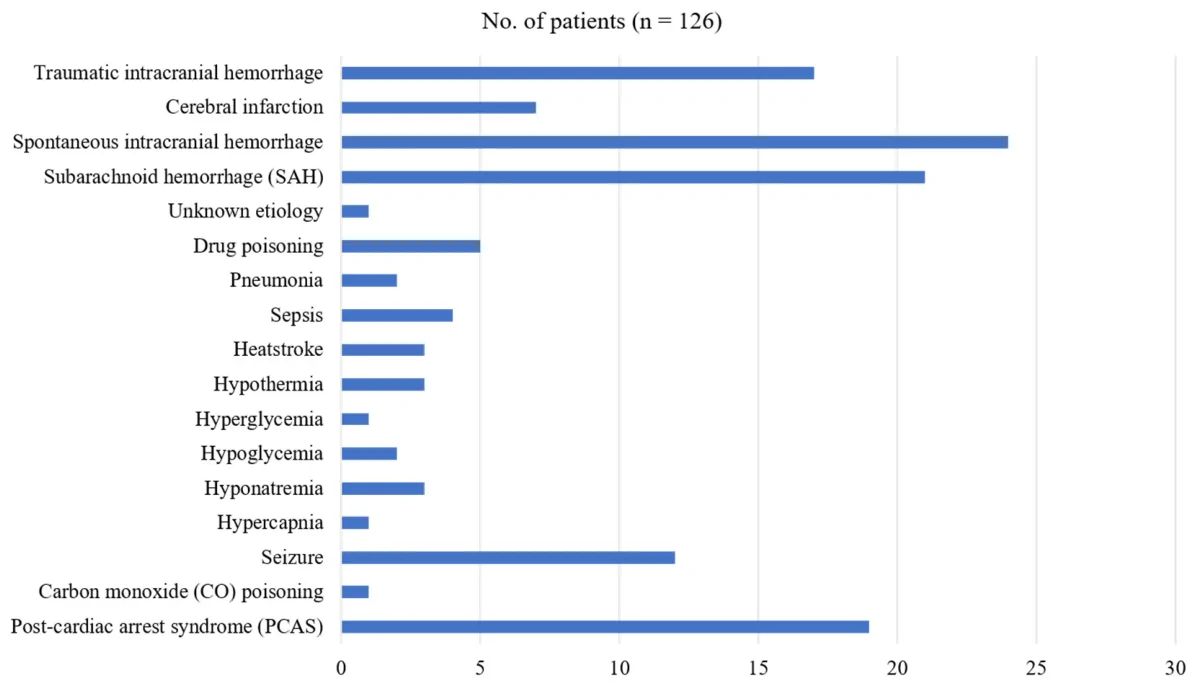
Etiology of patients in the study (n = 126).

Figure 2 shows etiologies. Among 126 patients, leading causes of impaired consciousness were spontaneous intracranial hemorrhage (24 patients, 19.0%), subarachnoid hemorrhage (21 patients, 16.7%), traumatic intracranial hemorrhage (17 patients, 13.5%), post–cardiac arrest syndrome (19 patients, 15.1%), and epileptic seizures (12 patients, 9.5%). Together, these accounted for 73.8% of patients; the remainder had diverse etiologies, including cerebral infarction, intoxication, metabolic disorders, and environmental emergencies, reflecting broad neurological and non-neurological origins.

### 3.2. Longitudinal Changes in Quantitative Pupillary Parameters in the Overall Cohort

**Figure 3:**
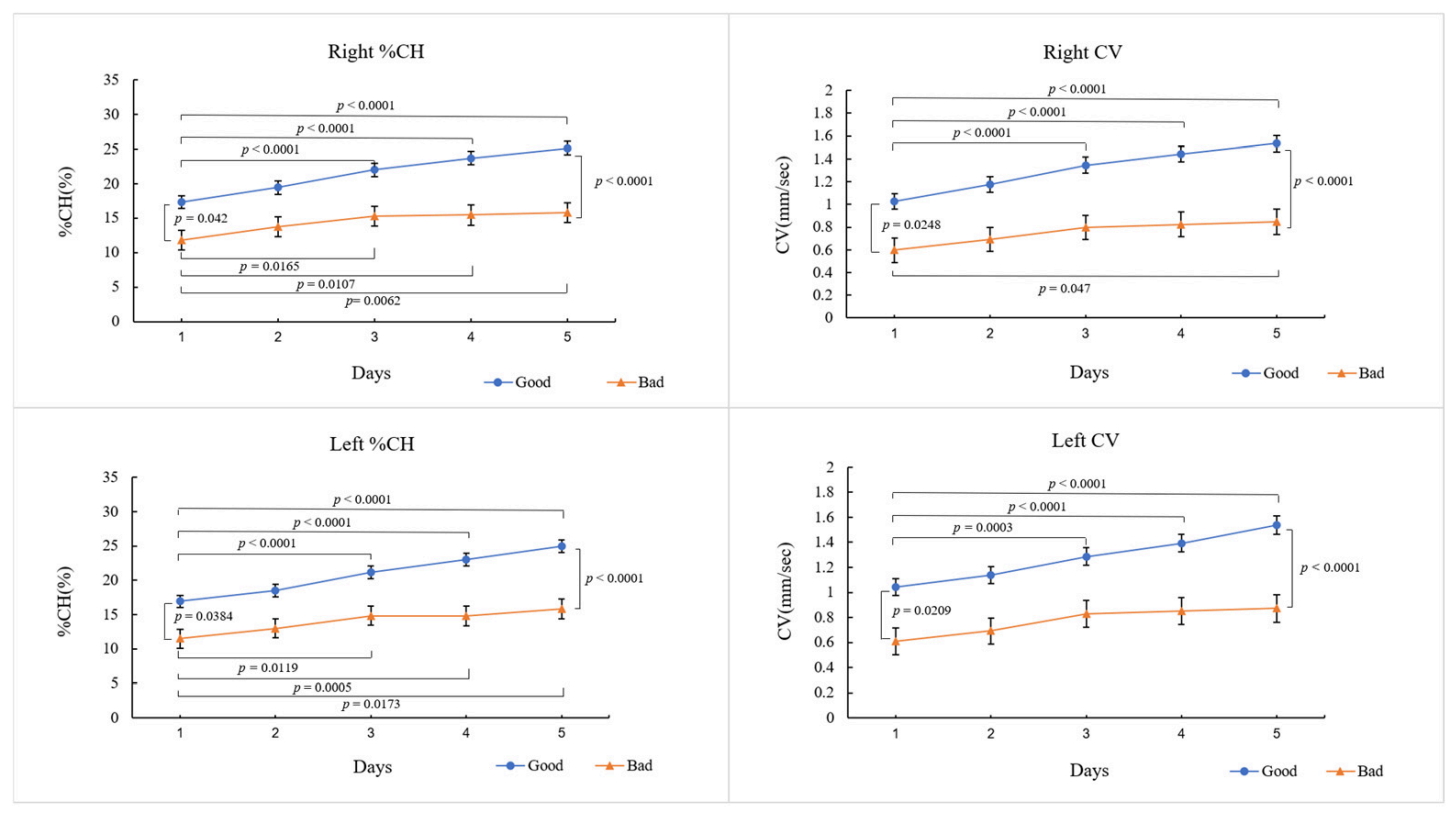
Comparison of the daily values of %CH and CV between the Good and Bad outcome groups in the study. Good, Good Outcome Group (CRS-R), including cases with EMCS and MCS; Bad, Bad Outcome Group, including cases with UWS and coma; %CH, percentage change in pupil diameter; CV, constriction velocity.

Figure 3 shows longitudinal changes in bilateral CV and %CH in the Good and Bad outcome groups and the post hoc multiple-comparison results. In the Good outcome group, CV and %CH continuously increased from day 1 to 5. Conversely, in the Bad out-come group, %CH plateaued after day 3, whereas CV exhibited only slight longitudinal increases (Figure 3).

### 3.3. Longitudinal Analysis

GLMM analysis demonstrated significant main effects of group and time on bilateral CV and %CH (all *p* < 0.0001, except for the group effect on left-eye CV, *p* = 0.0007, Table 3). To provide information on the magnitude and precision of the estimated associations, fixed-effect parameter estimates (β coefficients) and their corresponding 95% confidence intervals are presented in Supplementary Tables S2 and S3, using indicator coding and effect coding, respectively. Significant group-by-time interactions were observed for bilateral %CH (right eye, *p* = 0.0101, left eye, *p* = 0.0032), indicating significantly different %CH trajectories between the Good and Bad outcome groups. Conversely, no significant group-by-time interaction was observed for CV in either eye (right eye, *p* = 0.0531, left eye, *p* = 0.1231).

Graphical residual diagnostics showed that the conditional residuals were generally centered around zero. The central portions of the normal Q–Q plots were approximately consistent with normality, although deviations were observed in the tails. Residual-versus-fitted-value plots showed no pronounced systematic nonlinear patterns for %CH, whereas residual variability tended to increase with higher fitted values for CV, suggesting possible heteroscedasticity (Figures S2–S5).

Inspection of the conditional residual distributions revealed some asymmetry and outlying observations; therefore, minor deviations from the model assumptions cannot be excluded.

The number of pupillometry measurements per patient per day and the daily patient disposition, including reasons for discontinuation of measurements, are summarized in Supplementary Tables S4 and S1, respectively. The median number of pupillometry measurements per patient per day (interquartile range [IQR]) was 5 (3–9) on Day 1 and 12 (11–12) on each of Days 2, 3, 4, and 5. During the five-day observation period, 27 patients (21.4%) had incomplete longitudinal measurements, primarily due to ICU discharge or transfer to a general ward following clinical improvement (n = 23) or withholding of treatment associated with clinical deterioration (n = 4).

To assess the consistency of the primary findings among patients with complete longitudinal measurements, a complete-case sensitivity analysis was performed among the 99 patients with available pupillometry measurements across all five study days. The outcome-by-time interaction for %CH remained statistically significant in both eyes, with *p*-values of 0.0443 versus 0.0101 for the right eye and 0.0166 versus 0.0032 for the left eye in the complete-case and primary analyses, respectively (Table S5). These findings were broadly consistent with the primary analysis, although the complete-case analysis cannot exclude bias associated with informative dropout.

#### 3.3.1. Sensitivity Analyses of Longitudinal Outcomes

The results of sensitivity analyses adjusting for age, sex, underlying etiology, Glasgow Coma Scale (GCS) score at ICU admission (sum of the eye-opening and motor response components), targeted temperature management (TTM), and the use of continuous sedation and analgesia are presented in Supplementary Table S6. The main effect of neurological outcome group remained statistically significant for both percentage change in pupil diameter (%CH) and constriction velocity (CV) in both eyes across all adjusted models (all *p* < 0.001).

Regarding differences in longitudinal trajectories between neurological outcome groups, the outcome-by-time interaction for left-eye %CH remained statistically significant across all adjusted models. In contrast, the interaction for right-eye %CH was no longer statistically significant in the model adjusted for continuous analgesia (*p* = 0.0857).

#### 3.3.2. Patient Distribution Across Etiological Subgroups

The distribution of patients by neurological outcome within each etiological subgroup across the observation period is presented in Supplementary Table S7. Given the particularly small sample sizes in the post-cardiac arrest syndrome (PCAS) and nonstructural brain injury subgroups, all etiology-specific subgroup analyses were considered strictly exploratory and hypothesis-generating.

### 3.4. Etiology-Specific Analyses

#### 3.4.1. Structural Brain Injury

**Figure 4:**
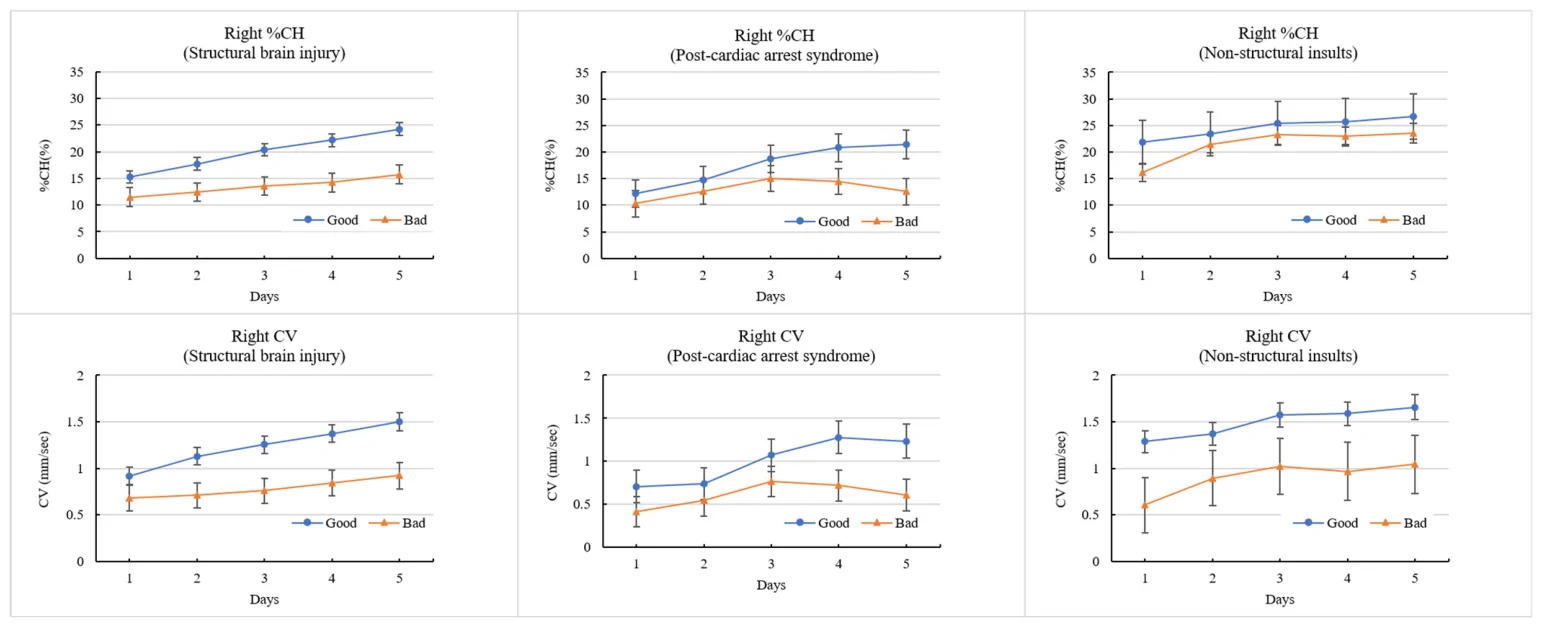
Comparison of the right-sided %CH and CV among the structural brain injury, post–cardiac arrest syndrome, and nonstructural brain injury groups. Abbreviations: Good, Good Outcome Group (CRS-R), including cases with EMCS and MCS; Bad, Bad Outcome Group, including cases with UWS and coma; %CH, percentage change in pupil diameter; CV, constriction velocity.

In patients with structural brain injury, GLMM analysis demonstrated significant group and time effects on bilateral CV and %CH (Table 3). However, no significant group-by-time interactions were observed for any pupillary parameter, indicating that no statistically significant differences were detected in the longitudinal trajectories of CV and %CH between the Good and Bad outcome groups. Figure 4 shows the corresponding trajectories.

#### 3.4.2. Post-Cardiac Arrest Syndrome

In post-cardiac arrest syndrome, GLMM showed significant time effects on bilateral CV and %CH (all *p* < 0.001). Only left-eye CV showed a significant group-by-time interaction (*p* = 0.0164); the remaining pupillary parameters showed no significant group-by-time interactions (Table 3). Figure 4 shows the corresponding longitudinal trajectories.

#### 3.4.3. Nonstructural Brain Injury

In patients with nonstructural brain injury, GLMM analysis showed a significant main effect of time only for right-eye %CH (*p* = 0.0292) and CV (*p* = 0.0451). No significant group effect or group-by-time interaction was observed for any pupillary parameter (Table 3). Figure 4 and Figure S1 show the corresponding longitudinal trajectories.

## 4. Discussion

### 4.1. Principal Findings and Comparison with Previous Studies

To our knowledge, few studies have characterized longitudinal trajectories of quantitative pupillary responses with serial automated pupillometry in a heterogeneous comatose ICU cohort [3,19].

GLMM analysis showed significantly different longitudinal %CH trajectories, whereas CV remained consistently higher in the Good outcome group without a significant interaction (Table 3). Throughout the observation period, the Good outcome group maintained higher values for both parameters, which increased over time. Conversely, %CH in the Bad outcome group increased from day 1 to day 3, then plateaued, yielding a significant group-by-time interaction (Figure 3). These findings suggest that early longitudinal changes in percentage change in pupil diameter (%CH) during the first three days after ICU admission may serve as a useful objective monitoring indicator reflecting the ongoing process of neurological recovery in comatose ICU patients.

Currently, only a limited number of studies have investigated the utility of quantitative pupillometry for neurological prognostication in patients with impaired consciousness. Most previous studies have focused on specific patient populations, particularly those with post–-ardiac arrest syndrome (PCAS) [7,8], or have evaluated pupillary parameters at a single time point during hospitalization [9]. Conversely, this study evaluated serial quantitative pupillary measurements throughout the early ICU stay, allowing the assessment of temporal trajectories rather than isolated values. Zarifkar et al. reported significantly lower CV and %CH values in patients with disorders of consciousness than in healthy controls [9]. Similarly, here, bilateral CV and %CH values were significantly higher in the Good outcome group than in the Bad outcome group (Table 3). Therefore, quantitative pupillometry may provide valuable complementary information for a more comprehensive assessment of evolving neurological status and the process of neurological recovery during the early phase of ICU hospitalization.

Suys et al. demonstrated in patients with PCAS that %CH was higher from an early stage and increased progressively over time in patients with favorable neurological outcomes, whereas it remained consistently low in those with unfavorable outcomes [7]. Consistently, this study demonstrated different longitudinal trajectories according to neurological outcomes, particularly for bilateral %CH (Table 3). These findings extend previous observations of patients with PCAS to a heterogeneous cohort of comatose patients in the ICU.

Taken together, these findings suggest that serial assessment of quantitative pupillary parameters may provide complementary information regarding neurological status during ICU hospitalization, although its added value over single-time-point measurements remains to be established.

### 4.2. Potential Neurophysiological Interpretation

Although the mechanisms linking the recovery of consciousness with quantitative pupillary responses remain incompletely understood, several neurophysiological pathways may provide a plausible framework for interpreting the present findings. Pupillary function is regulated by the balance between sympathetic and parasympathetic activity. Sympathetic activation induces pupillary dilation through the superior cervical ganglion and iris dilator muscle, whereas parasympathetic activity originating from the Edinger–Westphal nucleus mediates pupillary constriction through the ciliary ganglion and iris sphincter muscle [20,21,22]. Nonetheless, pupillary responses are not solely determined by the classical pupillary light reflex. Increasing evidence indicates that they are also modulated by the neural systems involved in arousal, attention, emotion, and higher-order cognitive processing [23].

Among these systems, the locus coeruleus (LC), a major noradrenergic nucleus involved in arousal and attention, is closely associated with pupillary dynamics. Murphy et al. demonstrated that pupil diameter covaries with blood oxygen level-dependent activity in the human LC, suggesting that pupillary responses reflect changes in LC activity associated with higher-order brain functions [24]. Furthermore, DiNuzzo et al. combined functional magnetic resonance imaging with eye-tracking and demonstrated that dynamic changes in pupil diameter are associated with coordinated activity involving the LC, anterior cingulate cortex, insular cortex, and other components of the salience network [25]. Additional evidence for the cortical modulation of pupillary responses comes from studies showing that healthy individuals exhibit pupillary constriction when viewing photographs of the sun despite the absence of actual luminance changes [26]. Similarly, Sangare et al. reported that subjective brightness-induced pupillary constriction may provide a window into residual cortical processing in patients with disorders of consciousness [27]. Overall, cortical processing alone can influence pupillary responses, independent of changes in retinal illumination. Thus, quantitative pupillary responses may be regulated through integrated interactions among autonomic, brainstem, subcortical, and cortical networks, rather than by any single neural structure.

Experimental studies have provided a possible framework for understanding how recovery may occur within these distributed networks. Gao et al. demonstrated in a rodent model that activation of the anterior nucleus gigantocellularis engaged multiple ascending arousal pathways and promoted emergence from pharmacologically induced coma [28]. These ascending projections relay through the thalamus and basal forebrain to the cerebral cortex, ultimately restoring cortical activity and behavioral responsiveness. If a similar hierarchical process occurs in humans, early recovery within the ascending arousal network may precede the restoration of higher-order cortical functions during the recovery of consciousness. Another, non-mutually exclusive explanation is that different brain regions exhibit varying susceptibilities to neurological injuries. Experimental studies have shown that neurons within the brainstem may be relatively more resistant to ischemic injury than neurons in the cerebral cortex, hippocampus, and striatum [29,30]. Such differential vulnerability may contribute to the temporal dissociation between recovery of quantitative pupillary responses and behavioral recovery of consciousness.

Clinical observations are consistent with the prognostic importance of preserved brainstem-mediated functions. The lack of wave V on auditory brainstem response following cardiac arrest has been associated with poor neurological outcomes, whereas automated pupillometry using the Neurological Pupil Index has demonstrated early prognostic utility in this population [31,32]. Here, the progressive increases in CV and %CH observed in the Good outcome group may reflect preservation or recovery of integrated autonomic and arousal-related networks. Conversely, the plateau in %CH observed after day 3 in the Bad outcome group may indicate a persistent dysfunction within these networks. Furthermore, pupillary values in the Good outcome group were comparable to previously reported reference values early during the ICU course, despite incomplete behavioral recovery of consciousness, which may be consistent with the possibility that recovery of brainstem-mediated pupillary function precedes full restoration of higher-order cortical function [33]. Nevertheless, age-related changes in pupillary responsiveness and the limited availability of population-specific reference values should be considered when interpreting these findings [34]. Importantly, these interpretations remain hypothetical, and this study did not establish a temporal or causal relationship between the recovery of pupillary responses and consciousness.

**Figure 5:**
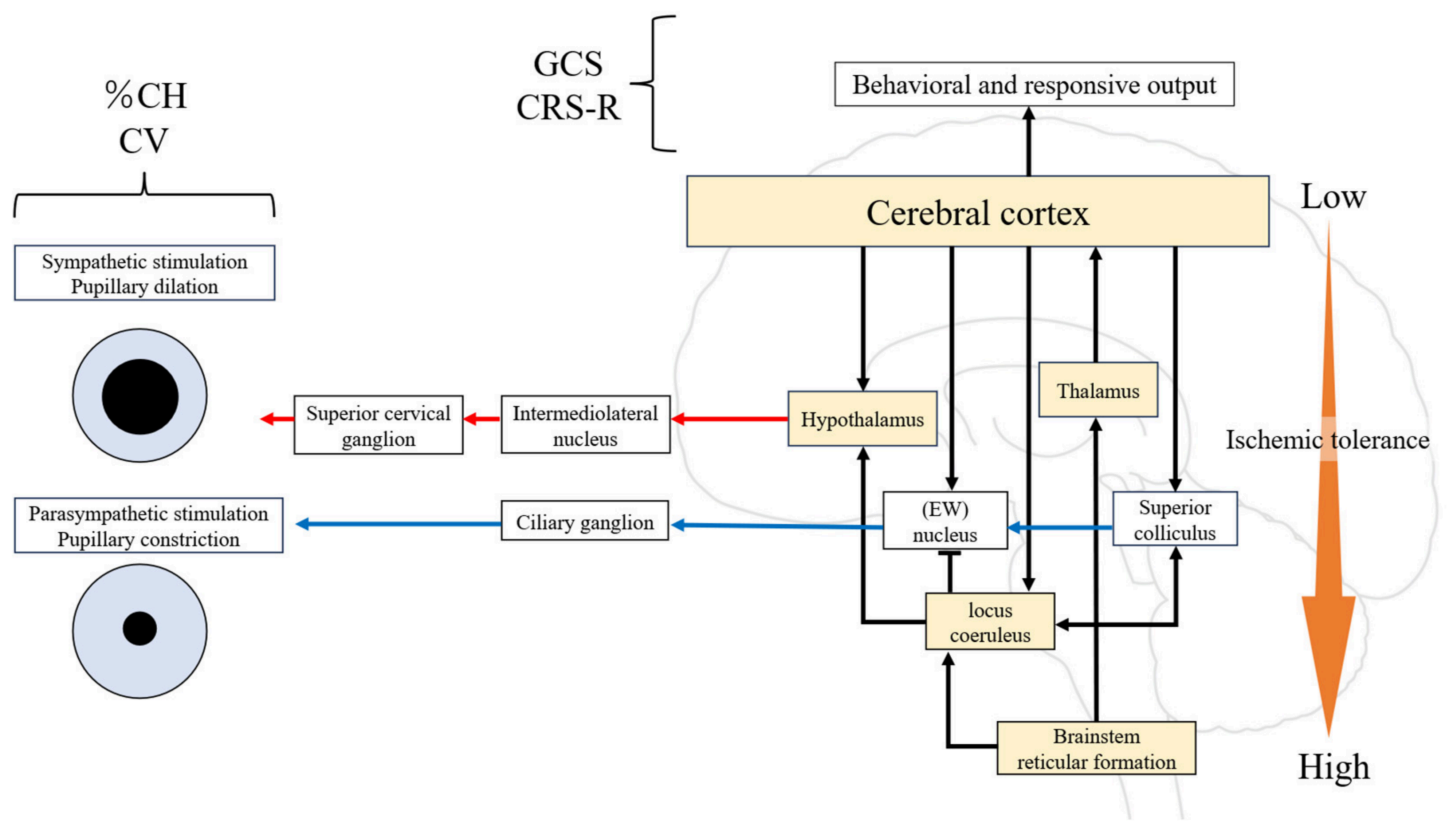
Schematic diagram of pupil control, consciousness and arousal, and brainstem ischemia tolerance. This figure presents a conceptual model of the potential neural interactions involved in the regulation of pupillary dynamics and does not imply a direct causal mechanism.

Figure 5 summarizes a proposed neurophysiological framework. Current anatomical, physiological, and clinical evidence illustrates how longitudinal quantitative pupillary changes may reflect recovery in distributed arousal-related networks during recovery of consciousness. The figure provides a conceptual model for interpreting the present findings rather than implying a direct causal mechanism. Future studies combining serial quantitative pupillometry with functional neuroimaging and electrophysiological assessments are necessary to validate this framework.

### 4.3. Clinical Implications of Longitudinal Quantitative Pupillometry

Most previous studies evaluating automated pupillometry have focused on measurements obtained at ICU admission or at a single predefined time point [9].

GLMM analysis demonstrated that the longitudinal trajectories of %CH differed significantly between the neurological outcome groups, whereas CV remained higher in the Good outcome group without a significant outcome-by-time interaction.

Therefore, serial measurements may provide information about changes in pupillary responses over time that cannot be captured by a single measurement.

Serial quantitative pupillometry offers practical advantages in neurocritical care. Its rapid, non-invasive, objective, highly reproducible measurements can be repeated throughout the ICU course without additional patient burden. Our research findings suggest that longitudinal assessment of quantitative pupillary parameters may complement multimodal neurological evaluations by providing objective information reflecting the ongoing process of consciousness recovery and dynamic changes in neurological status during the acute phase. Thus, serial assessment of pupillary dynamics may provide complementary objective information for monitoring changes in neurological status and persistent neurological dysfunction during the acute phase.

Quantitative pupillometry should not be regarded as a standalone prognostic tool but rather as a complementary component of established neurological assessments, including serial neurological examinations, the Coma Recovery Scale—Revised (CRS-R), electroencephalography (EEG), auditory brainstem responses (ABRs), neuroimaging, and other components of a multimodal prognostic assessment. Integrating longitudinal changes in quantitative pupillary parameters with these assessments may enhance multimodal neurological evaluation and provide a more comprehensive and precise characterization of evolving neurological status in comatose ICU patients.

Our findings suggest that serial quantitative pupillometry may provide complementary information for bedside neurological assessment. Prospective multicenter studies are warranted to validate these findings and determine whether longitudinal pupillary trajectories can be incorporated into multimodal prognostic algorithms for disorders of consciousness.

### 4.4. Limitations

This study had some limitations. First, this study was a small, single-center retrospective observational study. Given the retrospective observational design, the observed associations should not be interpreted as evidence of causal relationships between longitudinal changes in quantitative pupillary parameters and neurological recovery. Because the analysis was based on an existing cohort, no priori sample size calculation or statistical power analysis was performed. The relatively small sample size may have limited statistical power and the precision of the estimated effects, particularly for detecting subtle associations. Furthermore, the inclusion of comatose ICU patients with heterogeneous underlying etiologies warrants caution when interpreting and generalizing the findings. Larger, adequately powered, prospective multicenter studies are needed to confirm the reproducibility of the observed longitudinal changes in quantitative pupillary parameters and to establish their applicability to other patient populations.

An additional statistical limitation concerns the interpretation of multiple comparisons. Although numerous statistical tests were performed, including analyses of several pupillometry parameters and comparisons across time points, no formal adjustment for multiple comparisons was applied. In particular, the etiology-specific subgroup analyses were limited by relatively small sample sizes, especially among patients with post-cardiac arrest syndrome (n = 19) and nonstructural brain injury (n = 36), resulting in limited statistical power. Therefore, given the absence of formal adjustment for multiple comparisons and the constraints imposed by small subgroup sizes, these findings should be interpreted cautiously and regarded as exploratory and hypothesis-generating rather than confirmatory.

An additional statistical limitation concerns the assumptions underlying the Gaussian identity-link mixed-effects models. Graphical residual diagnostics revealed deviations from normality in the tails of the residual distributions and suggested possible heteroscedasticity, particularly for CV. These departures may affect the reliability of standard errors, confidence intervals, and statistical inference. Because alternative model specifications were not evaluated, the robustness of the findings to potential model misspecification remains uncertain. Accordingly, the results should be interpreted with caution.

Second, the potential effects of analgesic and sedative medications on quantitative pupillometry parameters and clinical assessments of consciousness cannot be completely excluded. Previous studies have reported that opioids and sedative agents may influence pupillary dynamics through their effects on the central nervous system [35,36]. Although an analgesia-first sedation strategy was adopted and additional sedative agents were administered with a target of light sedation (Richmond Agitation–Sedation Scale [RASS], −2 to 0), residual pharmacological effects on pupillary responses and behavioral assessments of consciousness may have persisted.

Furthermore, although continuous sedation and continuous analgesia were considered in the sensitivity analyses, these exposures were evaluated as binary variables indicating their presence or absence, rather than according to precise daily or cumulative drug doses or administration timing relative to individual pupillometry assessments. Notably, after adjustment for continuous analgesic administration, the outcome-by-time interaction for right-eye %CH was no longer statistically significant (*p* = 0.0857), whereas the corresponding interaction for left-eye %CH remained statistically significant (*p* = 0.0209) (Table S6). These findings suggest that the observed longitudinal associations, particularly for right-eye %CH, may be sensitive to adjustment for analgesic exposure. However, a change in statistical significance alone does not establish the magnitude or mechanism of medication-related confounding. Under these routine clinical sedation and analgesia practices, gradual improvements in %CH and CV were observed among patients with favorable neurological outcomes. Although these findings suggest that quantitative pupillometry may provide supplementary information regarding longitudinal changes in neurological status, the present study does not establish its clinical utility in the presence of potential medication-related confounding. Further investigations incorporating specific drug doses and administration timing are required to characterize the effects of individual sedative and analgesic agents on pupillary dynamics and to establish the validity of quantitative pupillometry as an objective monitoring indicator.

Third, the retrospective operational classification of consciousness states was subject to important methodological limitations. Standardized prospective assessments using the Coma Recovery Scale—Revised (CRS-R) were not performed. Instead, consciousness states were retrospectively classified using Glasgow Coma Scale (GCS) sub-scores recorded every two hours and detailed clinical documentation, according to operational definitions adapted from previously published criteria [15].

Although two board-certified specialists in emergency and critical care medicine independently reviewed the clinical records and resolved disagreements through consensus, the GCS itself does not include certain behavioral assessments essential for distinguishing vegetative state/unresponsive wakefulness syndrome (VS/UWS) from minimally conscious state (MCS), such as visual pursuit, as highlighted by Bodien et al. Therefore, the GCS and classifications derived from it have inherent limitations in accurately determining the level of consciousness.

In addition, retrospective reliance on routine clinical documentation may have resulted in the omission of subtle or intermittent behavioral signs of consciousness, including transient visual pursuit or intermittent command following. Consequently, outcome misclassification cannot be excluded, particularly the possibility that patients with subtle signs of MCS were classified as having UWS because sufficient evidence of conscious behavior was not documented.

Furthermore, the neurological endpoint in this study reflected the best documented level of consciousness attained during ICU hospitalization. Long-term functional or neurological outcomes, including the Cerebral Performance Category (CPC), Glasgow Outcome Scale–Extended (GOSE), modified Rankin Scale (mRS), and post-discharge mortality, were not collected or assessed at predefined follow-up time points. Accordingly, the findings should be interpreted strictly as associations between longitudinal changes in quantitative pupillary parameters and consciousness recovery during ICU hospitalization, rather than as evidence of long-term prognostic performance. Prospective studies incorporating serial standardized CRS-R assessments and long-term functional follow-up are needed to characterize more precisely the relationship between longitudinal pupillary dynamics and different stages of neurological recovery.

Fourth, the exclusion of patients who died within five days of ICU admission represents a major limitation of the longitudinal study design and may have introduced selection or survivor bias. A total of 35 patients (20.5%) were excluded because of early death. In routine clinical practice, patients with profound hemodynamic instability and an anticipated high likelihood of imminent death, including those with post-cardiac arrest syndrome following prolonged cardiac arrest, catastrophic stroke, or severe traumatic brain injury, often required prioritization of life-saving interventions, essential clinical care, or end-of-life care over frequent quantitative pupillometry measurements.

Consequently, sufficient longitudinal pupillometry data were generally unavailable for these patients, many of whom exhibited clinically observed bilateral fixed and dilated pupils at initial assessment. Excluding this extremely critically ill subgroup may have restricted the spectrum of disease severity represented in the analyzed cohort.

Depending on how the unobserved pupillary trajectories of these excluded patients differed from those of patients with poor neurological outcomes included in the analysis, the magnitude of the observed longitudinal differences between outcome groups may have been either overestimated or underestimated. The direction and magnitude of this potential bias remain uncertain. Therefore, the findings may be more applicable to comatose ICU patients who survived the initial acute period than to the entire spectrum of critically ill comatose patients. Caution is particularly warranted when extrapolating these longitudinal patterns to patients at imminent risk of brain death or fatal cerebral herniation.

Fifth, incomplete longitudinal observations and potentially informative dropout represent additional limitations. During the five-day observation period, 27 of the 126 patients (21.4%) had incomplete longitudinal pupillometry data. Of these, 23 patients discontinued measurements following clinical improvement and ICU discharge or transfer to a general ward, whereas four discontinued measurements following decisions to withhold life-sustaining treatment or transition to end-of-life care (Table S1).

Because these reasons for dropout were closely related to patients’ clinical courses, the missing-data mechanism may have been informative and potentially missing not at random (MNAR), thereby violating the missing-at-random (MAR) assumption underlying the primary mixed-model analysis. Such missingness may have biased the estimated longitudinal trajectories, particularly toward the end of the observation period.

To assess the robustness of the primary findings, an additional complete-case sensitivity analysis was performed among the 99 patients with available measurements for all five study days. As shown in Table S5, the outcome-by-time interaction remained statistically significant for both right-sided %CH (*p* = 0.0443 in the complete-case analysis versus *p* = 0.0101 in the primary analysis) and left-sided %CH (*p* = 0.0166 versus *p* = 0.0032, respectively). The overall longitudinal patterns of CV were also generally consistent between the analyses.

These findings suggest that the principal longitudinal associations were not solely attributable to incomplete observations. Nevertheless, complete-case analysis cannot eliminate bias arising from an MNAR mechanism, and the possibility of residual bias related to informative dropout remains. Therefore, the results should be interpreted with appropriate caution.

Sixth, the proposed neurophysiological mechanisms underlying the observed longitudinal changes in quantitative pupillary parameters remain hypothetical. Although previous anatomical, physiological, experimental, and clinical evidence provides a rationale for the proposed relationships, serial quantitative pupillometry alone cannot identify the specific neural structures or pathways responsible for the observed changes. Future studies integrating quantitative pupillometry with functional neuroimaging, electrophysiological assessments, and other complementary neurological assessment modalities are required to investigate these potential mechanisms.

Finally, the heterogeneity of underlying etiologies may have influenced the observed longitudinal pupillometry patterns. Although the inclusion of patients with diverse causes of impaired consciousness may enhance the clinical relevance of the findings, different pathophysiological mechanisms may contribute differently to changes in quantitative pupillary responses over time. Therefore, adequately powered, prospective, etiology-specific studies are needed to clarify these relationships and determine whether the observed longitudinal associations are consistent across different causes of impaired consciousness.

## 5. Conclusions

Serial quantitative pupillometry demonstrated that longitudinal changes in CV and %CH were associated with the best documented level of consciousness achieved during ICU hospitalization in a heterogeneous cohort of comatose patients. These findings suggest that serial pupillometry may complement clinical assessments of consciousness recovery, although its long-term prognostic value remains to be established.

## Figures and Tables

**Table 1:** GCS-Based Operational Criteria for Retrospective Classification of Consciousness States.

EMCS	A state in which consistent communication or the functional and appropriate use of objects is possible. While the GCS criteria generally require M = 6 and V = 4–5, in intubated patients, cases where communication via writing or gestures was confirmed were also included.
MCS	A state in which M ≥ 5, indicating compliance with commands or localization of pain. Furthermore, even if the M score was 4 or lower, cases were classified as MCS based on clinical judgment if eye tracking, sustained fixation, or purposeful behavior in response to specific stimuli were confirmed in the medical records.
UWS	A state in which spontaneous or stimulus-induced eye opening (E > 1) is observed, but purposeful behavior such as compliance with commands, localization of pain, or eye tracking is not observed. Furthermore, cases that did not meet the above criteria were also classified as UWS.
Coma	A state in which the patient does not open the eyes (awaken) even in response to strong external stimuli (E = 1) and does not exhibit purposeful motor responses (M ≤ 4). This allowed for evaluation distinct from UWS, which involves spontaneous eye opening.

Note: The consciousness categories presented in this table represent study-specific operational classifications based on retrospectively reviewed GCS subscores and documented clinical behavioral responses. These classifications were not established through formal CRS-R assessments and should not be considered equivalent to standardized CRS-R diagnoses. In particular, a GCS motor response score of 5 (localization of painful stimuli) alone does not fulfill the diagnostic criteria for MCS.

**Table 2:** Comparison of characteristics between the Good and Bad outcome groups.

Factors	All (n = 126)	Good (n = 88)	Bad (n = 38)	*p*-Value
(A) Parameters				
Age, years	70.5 (54.0–80.0)	65.0 (53.3–76.8)	77.0 (60.3–84.3)	0.0143
Male sex, n (%)	75 (59.5)	59 (67.0)	16 (42.1)	0.008
Time to best observed state or final assessment, hours	68.0 (15.0–148.0)	74.0 (30.6–137.6)	106.0 (52.5–195.3)	0.0368
(B) Consciousness Categories				
EMCS (n)	40	40	0	—
MCS (n)	48	48	0	
UWS (n)	20	0	20	
COMA (n)	18	0	18	
(C) Etiology of Impaired Consciousness				
Post-cardiac arrest syndrome, n (%)	19 (15.1)	9 (10.2)	10 (26.3)	0.0098
Structural brain injury, n (%)	71 (56.3)	48 (54.5)	23 (60.5)	
Non-structural insults, n (%)	36 (28.6)	31 (35.2)	5 (13.2)	

Abbreviations: Good, Good Outcome Group (CRS-R), including cases with EMCS and MCS; Bad, Bad Outcome Group, including cases with UWS and coma; %CH, percentage change in pupil diameter; CV, pupillary constriction velocity. Data are expressed as number (percentage) or median (interquartile range). The *p*-value for the etiology section is an omnibus *p*-value calculated using Pearson’s chi-square test across all three etiology categories.

**Table 3:** Results of the generalized linear model (GLMM) for the daily trends in quantitative pupillary indices over time in the “Good” and ‘Bad’ groups within each of the three subgroups Structural Brain Injury Group, PCAS Group, and Non-Structural Disease Group classified according to clinical features.

		Day 1		Day 2		Day 3		Day 4		Day 5		GLMM (*p*-Value)		
	Outcome	Good	Bad	Good	Bad	Good	Bad	Good	Bad	Good	Bad	Time	Group	Inter.
Variable/Etiology														
Right %CH (%)														
All subjects		17.30 ± 0.93	11.77 ± 1.43	19.43 ± 0.93	13.71 ± 1.43	21.98 ± 0.95	15.25 ± 1.43	23.70 ± 0.95	15.44 ± 1.44	25.12 ± 0.97	15.79 ± 1.48	<0.0001	<0.0001	0.0101
Structural		14.46 ± 1.28	9.78 ± 2.01	17.41 ± 1.27	11.45 ± 1.99	19.92 ± 1.27	12.85 ± 1.99	22.09 ± 1.27	13.95 ± 1.99	23.91 ± 1.28	14.88 ± 1.99	<0.0001	0.0019	0.0534
PCABI		12.22 ± 2.92	10.82 ± 2.58	13.88 ± 2.92	13.31 ± 2.58	17.66 ± 2.92	16.39 ± 2.58	20.44 ± 2.92	15.86 ± 2.58	20.97 ± 2.92	13.44 ± 2.58	0.0003	0.3726	0.1662
Non-structural		20.71 ± 2.21	18.27 ± 4.82	22.23 ± 2.21	22.91 ± 4.82	24.09 ± 2.23	25.27 ± 4.82	24.56 ± 2.21	24.56 ± 4.82	25.60 ± 2.21	25.14 ± 4.82	0.0292	0.965	0.9128
Left %CH (%)														
All subjects		16.92 ± 0.90	11.50 ± 1.39	18.48 ± 0.90	12.99 ± 1.39	21.13 ± 0.92	14.83 ± 1.39	23.04 ± 0.92	14.79 ± 1.40	24.94 ± 0.94	15.86 ± 1.43	<0.0001	<0.0001	0.0032
Structural		14.28 ± 1.26	9.03 ± 1.98	16.97 ± 1.26	10.80 ± 1.98	19.30 ± 1.26	13.34 ± 1.98	21.11 ± 1.26	14.11 ± 1.98	23.29 ± 1.26	15.49 ± 1.98	<0.0001	0.004	0.4807
PCABI		13.00 ± 2.67	11.01 ± 2.42	14.28 ± 2.67	14.09 ± 2.36	17.68 ± 2.67	16.94 ± 2.36	20.88 ± 2.67	15.41 ± 2.36	23.08 ± 2.67	15.06 ± 2.36	0.0001	0.2889	0.0662
Non-structural		19.57 ± 1.99	21.40 ± 4.33	19.82 ± 1.99	20.73 ± 4.33	22.00 ± 2.00	22.67 ± 4.33	24.49 ± 1.99	23.56 ± 4.33	25.49 ± 1.99	22.28 ± 4.33	0.0828	0.9724	0.6259
Right CV (mm/s)														
All subjects		1.03 ± 0.07	0.60 ± 0.11	1.18 ± 0.07	0.69 ± 0.11	1.35 ± 0.07	0.80 ± 0.11	1.44 ± 0.07	0.82 ± 0.11	1.54 ± 0.07	0.85 ± 0.11	<0.0001	<0.0001	0.0531
Structural		0.87± 0.10	0.60 ± 0.15	1.12 ± 0.10	0.65 ± 0.15	1.22 ± 0.10	0.73 ± 0.15	1.32 ± 0.10	0.82 ± 0.15	1.46 ± 0.10	0.88 ± 0.15	<0.0001	0.0052	0.1643
PCABI		0.75 ± 0.22	0.43 ± 0.20	0.68 ± 0.22	0.57 ± 0.20	0.99 ± 0.22	0.83 ± 0.20	1.25 ± 0.22	0.79 ± 0.20	1.21 ± 0.22	0.65 ± 0.20	0.0003	0.2393	0.2032
Non-structural		1.24 ± 0.17	0.71 ± 0.36	1.28 ± 0.17	1.03 ± 0.36	1.48 ± 0.17	1.17 ± 0.36	1.50 ± 0.17	1.08 ± 0.36	1.58 ± 0.17	1.16 ± 0.36	0.0451	0.2868	0.879
Left CV (mm/s)														
All subjects		1.04 ± 0.07	0.61 ± 0.11	1.14 ± 0.07	0.69 ± 0.10	1.28 ± 0.07	0.83 ± 0.10	1.39 ± 0.07	0.85 ± 0.11	1.54 ± 0.07	0.87 ± 0.11	<0.0001	0.0007	0.1231
Structural		0.87 ± 0.09	0.51 ± 0.15	1.11 ± 0.09	0.63 ± 0.15	1.16 ± 0.09	0.79 ± 0.15	1.24 ± 0.09	0.82 ± 0.15	1.41 ± 0.09	0.88 ± 0.15	<0.0001	0.0052	0.6827
PCABI		0.78 ± 0.23	0.43 ± 0.21	0.72 ± 0.23	0.61 ± 0.20	1.02 ± 0.23	0.91 ± 0.20	1.39 ± 0.23	0.83 ± 0.20	1.56 ± 0.23	0.77 ± 0.20	<0.0001	0.1728	0.0164
Non-structural		1.23 ± 0.16	0.93 ± 0.34	1.18 ± 0.16	0.98 ± 0.34	1.35 ± 0.16	1.13 ± 0.34	1.49 ± 0.16	1.14 ± 0.34	1.58 ± 0.16	1.03 ± 0.34	0.2476	0.3441	0.7522

Abbreviations: Good, good outcome group (CRS-R), including cases with EMCS and MCS; Bad, bad outcome group, including cases with UWS and coma; %CH, percentage change in pupil diameter; CV, pupillary constriction velocity.

## Data Availability

The data supporting the findings of this study are available from the corresponding author upon request.

## Supplementary Materials

The following supporting information can be downloaded at: https://www.mdpi.com/article/10.3390/diagnostics16213483/s1, Figure S1: Longitudinal trajectories of Left %CH and Left CV across Days 1–5 among the structural brain injury, post-cardiac arrest syndrome (PCAS), and non-structural brain insult groups, stratified by neurological outcome; Figure S2: Validation of model assumptions (homoscedasticity and normality of conditional residuals) for the Generalized Linear Mixed Model of Right %CH; Figure S3: Validation of model assumptions (homoscedasticity and normality of conditional residuals) for the Generalized Linear Mixed Model of Right CV; Figure S4: Validation of model assumptions (homoscedasticity and normality of conditional residuals) for the Generalized Linear Mixed Model of Left %CH; Figure S5: Validation of model assumptions (homoscedasticity and normality of conditional residuals) for the Generalized Linear Mixed Model of Left CV; Table S1: Daily patient disposition and clinical reasons for data discontinuation stratified by neurological outcome; Table S2: Parameter estimates of fixed effects from Generalized Linear Mixed Models (GLMMs) for quantitative pupillometry parameters (%CH and CV) using reference coding; Table S3: Parameter estimates of fixed effects from Generalized Linear Mixed Models (GLMMs) for quantitative pupillometry parameters (%CH and CV) using effect coding; Table S4: Daily distribution of the number of pupillometry measurements per patient (Days 1–5); Table S5: Fixed-effects test results of Generalized Linear Mixed Models (GLMMs) for pupillometry parameters (%CH and CV): Primary cohort (n = 126) vs. Complete-case cohort (n = 99); Table S6: Sensitivity analyses for longitudinal pupillary measurements using Generalized Linear Mixed Models (GLMMs) adjusted for potential confounders and clinical interventions; Table S7: Daily sample sizes of etiology-based subgroups stratified by neurological outcome.

## Abbreviations

The following abbreviations are used in this manuscript:

AUC	Area under the curve
CI	Confidence interval
COMA	Coma
CRS-R	Coma Recovery Scale—Revised
CV	Constriction velocity
EMCS	Emerged from the minimally conscious state
GCS	Glasgow Coma Scale
GLMM	Generalized linear mixed model
ICU	Intensive care unit
LC	Locus coeruleus
MCS	Minimally conscious state
NPi	Neurological Pupil Index
PCAS	Post-cardiac arrest syndrome
%CH	Percentage change in pupil diameter
RASS	Richmond Agitation-Sedation Scale
ROC	Receiver operating characteristic
UWS	Unresponsive wakefulness syndrome
